# Case report: Hypoparathyroidism-sensorineural hearing loss-renal dysplasia without febrile seizures: a novel mutation in the GATA3 gene

**DOI:** 10.3389/fendo.2025.1502545

**Published:** 2025-02-12

**Authors:** Haibin Chen, Yudi Zhang, Xueyao Yang, Yongzhen Li

**Affiliations:** ^1^ Department of Endocrinology, Changsha Hospital of Hunan Normal University, Changsha, Hunan, China; ^2^ Department of Endocrinology, The Fourth Hospital of Changsha, Changsha, Hunan, China; ^3^ Department of Pediatrics, The Second Xiangya Hospital, Central South University, Changsha, Hunan, China

**Keywords:** HDR syndrome, GATA3 gene, GATA3 mutation, novel mutation, pediatric

## Abstract

**Objective:**

This study aims to summarize the diagnostic and treatment experience of a case of Hypoparathyroidism-Sensorineural Hearing Loss-Renal Dysplasia (HDR) syndrome caused by a heterozygous mutation in the GATA3 gene.

**Methods:**

The diagnostic and treatment process of the patient with HDR syndrome in our hospital was compared and analyzed.

**Results:**

A 9-month-old male infant with a history of poor physical condition and increased susceptibility to infections. At the age of 2 months, ptosis was observed in the left eye. Laboratory tests revealed decreased serum calcium, elevated blood phosphorus levels, and reduced parathyroid hormone (PTH) levels, indicating the presence of “Hypoparathyroidism”. Genetic testing identified a heterozygous mutation in the GATA3 gene in the patient, specifically a nucleotide change from G to T at position 800 (c.800G>T). This mutation resulted in the substitution of cysteine with phenylalanine at amino acid position 267 (p.C267F). The missense mutation was determined to be both pathogenic and novel.

**Conclusion:**

Early genetic testing should be prioritized, and regular monitoring of kidney development and hearing status is essential. The reported case, featuring the novel GATA3 gene mutation c.800G>T (p.C267F), contributes to the enrichment of the genetic database.

## 1 Introduction

Hypoparathyroidism-Sensorineural Hearing Loss-Renal Dysplasia syndrome (HDR syndrome), also known as Barakat syndrome, is a rare autosomal dominant genetic disorder (1). HDR syndrome is primarily characterized by hypocalcemia-induced increased neuromuscular excitability, such as non-febrile seizures, muscle spasms, and myalgia (2). It is associated with hearing loss and abnormal kidney structure or function, caused by mutations in the GATA3 gene located on chromosome 10p14 (3, 4). The GATA3 gene belongs to the dual zinc finger transcription factor family and consists of 6 exons that encode 444 amino acids, including 2 transactivation domains and 2 zinc finger domains (5). This gene plays a crucial role in embryonic development in vertebrates and is expressed in the developing parathyroid gland, inner ear, and kidneys (6). The incidence of HDR syndrome is still clear, with limited reports on the clinical phenotype and genotype of HDR syndrome patients in China. In this case, the patient had a novel heterozygous mutation in the GATA3 gene, C.800G>T (p.C267F), which has not been reported before. Pathogenic analysis suggests that this mutation can lead to amino acid changes and is considered pathogenic. Therefore, this study aims to investigate the clinical features and genotypes of HDR syndrome, providing a foundation for accurate diagnosis and improving our understanding of the syndrome. This research will contribute to early screening and diagnosis, reducing misdiagnosis and missed diagnoses.

## 2 Case report

### 2.1 General information

A 9-month-old male infant, with a height of 72cm and a weight of 10Kg, was admitted to our hospital for the second time due to “recurrent fever for 3 days”. He exhibited a history of a weakened constitution. At 2 months of age, the patient presented with ptosis in the left eye (excluding myasthenia gravis). At 4 months of age, the patient was diagnosed with “viral encephalitis” and had a history of febrile seizures. An examination revealed the presence of a patent foramen ovale. At 7 months of age, the patient was admitted to our hospital due to recurrent symptoms of fever, cough, and wheezing. There is no history of hepatitis, known exposure, surgery, trauma, or blood transfusion. The patient has allergies to amoxicillin, cefadroxil, or cefuroxime. Vaccinations are up to date. There is no significant personal or family medical history.

In the ten days preceding this hospitalization, the patient experienced four episodes of breath-holding spells characterized by sudden breath-holding and cyanosis while crying. These episodes did not coincide with fever. During a prior hospitalization at another institution, the patient also exhibited bilateral thumb adduction and stiffness in the right lower limb, without tremors. However, the patient remained conscious and responsive to the mother’s call, and the symptoms improved with calcium supplementation.

### 2.2 Diagnosis and treatment process

**Figure 1:**
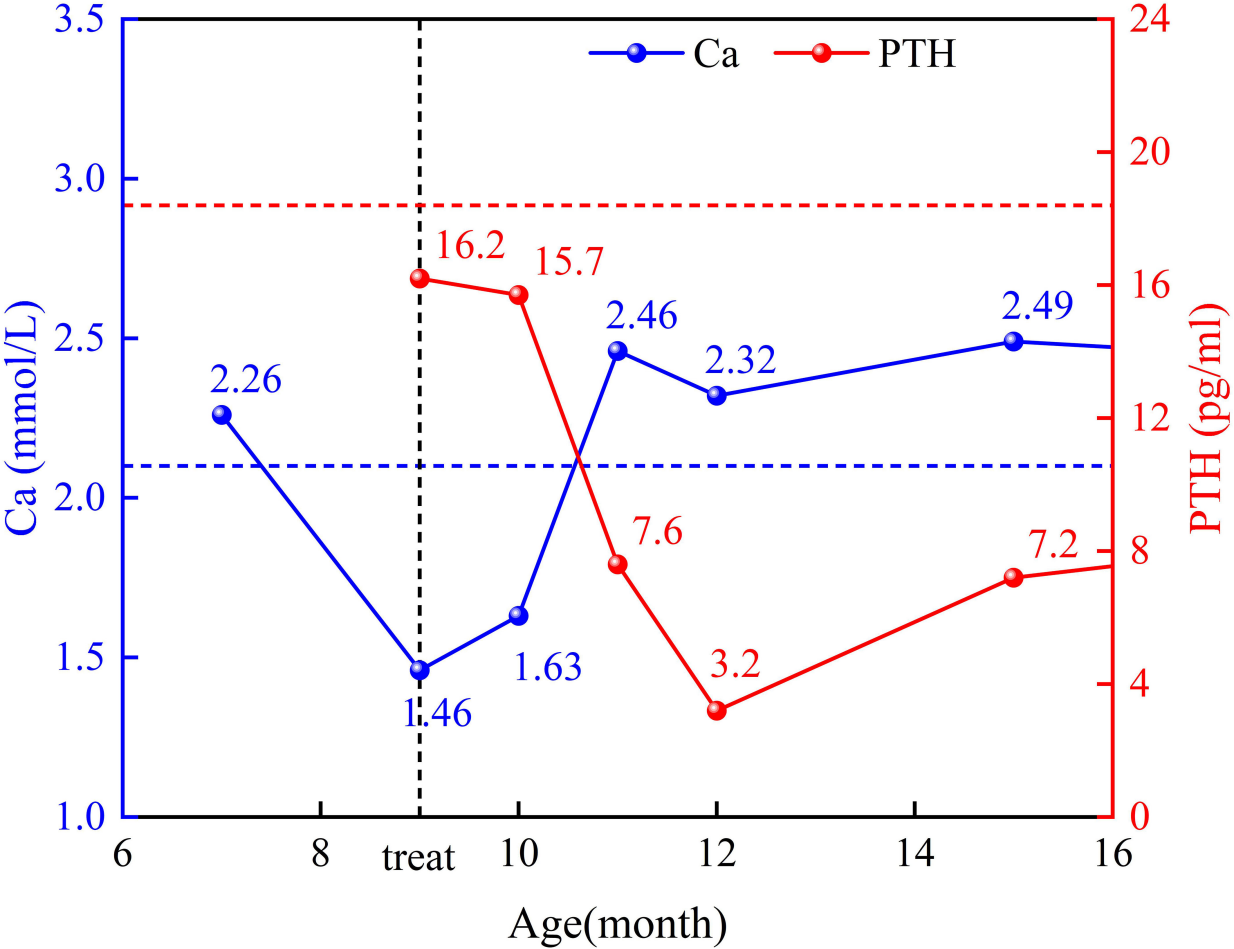
The trend of blood calcium and PTH levels changes in the patient.

During the patient’s first hospitalization at 7 months old, the blood calcium level was normal, but during the current hospitalization, there was a decrease in serum calcium levels and an increase in blood phosphorus levels. Low levels of parathyroid hormone (PTH) were observed, indicating “hypoparathyroidism.” All other hormone levels were within the normal range. Transient proteinuria was observed (+). **Table 1** presents the results of the biochemical examination during this hospitalization, while **Figure 1** illustrates the trends in blood calcium and PTH levels over the patient’s age. Cardiac ultrasound revealed no abnormalities in cardiac structure or blood flow. Abdominal ultrasound, which examined the liver, gallbladder, pancreas, spleen, kidneys, and retroperitoneum, showed no significant abnormalities. Ultrasound of the parathyroid, thyroid, and cervical lymph nodes revealed multiple lymph nodes in both sides of the neck, with enlarged lymph nodes in the right II region. However, no apparent abnormalities were found in the thyroid or parathyroid. Head magnetic resonance imaging (MRI) with contrast showed no abnormalities. Regarding the patient’s hearing examination, the infant passed the hearing screening (Otoacoustic Emissions test and Automated Auditory Brainstem Response test) at birth. During the hospitalization, the bilateral Auditory Brainstem Response (ABR) test was conducted and no abnormal electrophysiological changes were observed. Further genetic testing identified a heterozygous mutation in the GATA3 gene. The mutation occurred at nucleotide 800, where G was replaced by T (c.800G>T), resulting in the substitution of cysteine with phenylalanine at amino acid position 267 (p.C267F), indicating a missense mutation. No variations were detected in the same position in either the father or mother.

**Table 1:** The results of the biochemical examination of the patient during this hospitalization.

Index		Result	Normal range
Immunoglobulin	IgG	8.32	7.00~16.00(g/l)
	IgA	0.70	0.70~4.00(g/l)
	IgM	1.10	0.40~2.30(g/l)
Blood routine	WBC	13.39	5.00~12.00(10^9/L)
	HB	113	110~160(g/L)
	RBC	0.18	0.10~0.40(10^12/L)
	PLT	432	100~400 (10^9/L)
	LYM	5.10	1.10~3.20(10^9/L)
Biochemistry	AST	41.5	15.0~40.0(U/L)
	ALT	18.6	9.0~50.0(U/L)
	Cr	30.0	44.0~133.0(μmol/L)
	BUN	2.80	2.90~7.14(mmol/L)
Thyroid function	FT3	2.72	1.58~3.91(pg/ml)
	FT4	1.12	0.70~1.48(ng/dl)
	TSH	3.28	0.35~4.49(uIU/ml)
	A-TG	0.426	0.000~4.110(Iu/ml)
	A-TPO	0.403	0.000~5.610(Iu/ml)
Urinalysis	Urine protein	negative	negative

WBC, White blood cell; HB, Hemoglobin; RBC, Red blood cell; PLT, Platelet; LYM, Lymphocyte; AST, Aspertate Aminotransferase; ALT, Glutamic-pyruvic transaminase; CERA, Creatinine; BUN, Blood urea nitrogen; FT3, Free Triiodothyronine; FT4, Free thyroxine, TSH, Thyroid-stimulating hormone; A-TG, Anti-thyroglobulin antibodies, A-TPO, Anti-thyroid peroxidase.

After admission, the patient was treated with anti-infective, antitussive, bronchodilator, intravenous calcium supplements, and oral calcitriol, and showed improvement upon discharge. Following discharge, the child was regularly monitored for blood calcium and parathyroid hormone (PTH) levels at the outpatient clinic, and the oral calcitriol dosage was dynamically adjusted based on the results. Currently, the blood calcium levels are maintained within the normal range, and the parathyroid hormone levels have improved compared to before. (see **Figure 1**).

### 2.3 Genetic testing

In order to investigate the underlying cause of the patient’s recurrent pneumonia, wheezing, episodes of breath-holding without fever, hypoparathyroidism, hypocalcemia, and isolated ptosis, whole-exome gene sequencing and copy number variation analysis were performed.

**Figure 2:**
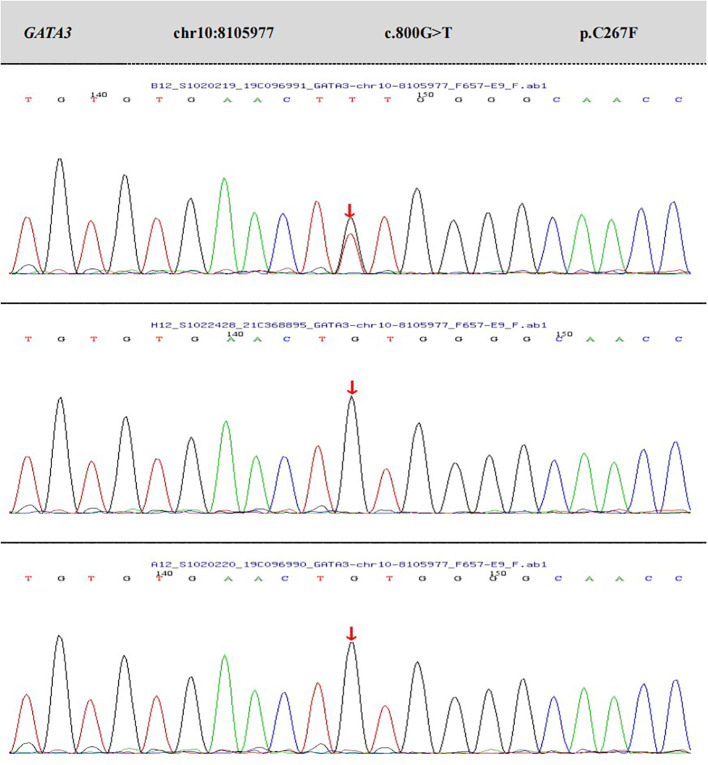
The results of peripheral blood whole-exome sequencing and CNV analysis, as well as Sanger validation, for the patient and their parents.

The genetic testing results (depicted in **Figure 2**) disclosed a heterozygous mutation within the GATA3 gene, specifically at nucleotide position 800 (located in exon 4; reference sequence: NM_001002295), where guanine (G) was substituted by thymine (T). This mutation results in an amino acid alteration from cysteine to phenylalanine at position 267 (designated as p.C267F).According to the latest ACMG guidelines, this variant is provisionally classified as a likely pathogenic variant based on the following criteria:PS2: The variant was confirmed to be *de novo* through family segregation analysis, as no variations were found in the same position in either the father or mother; PM2_Supporting: The variant is rare in the general population, as indicated by its frequency (-) in the normal population database.PP3: Bioinformatics protein function prediction software REVEL predicts the variant to be deleterious. Additionally, SIFT, PolyPhen_2, MutationTaster, and GERP+ also predict the variant to be deleterious. There are no relevant reports on this specific position in the literature database, and the ClinVar database does not provide any pathogenic analysis results for this variant.

**Figure 3:**
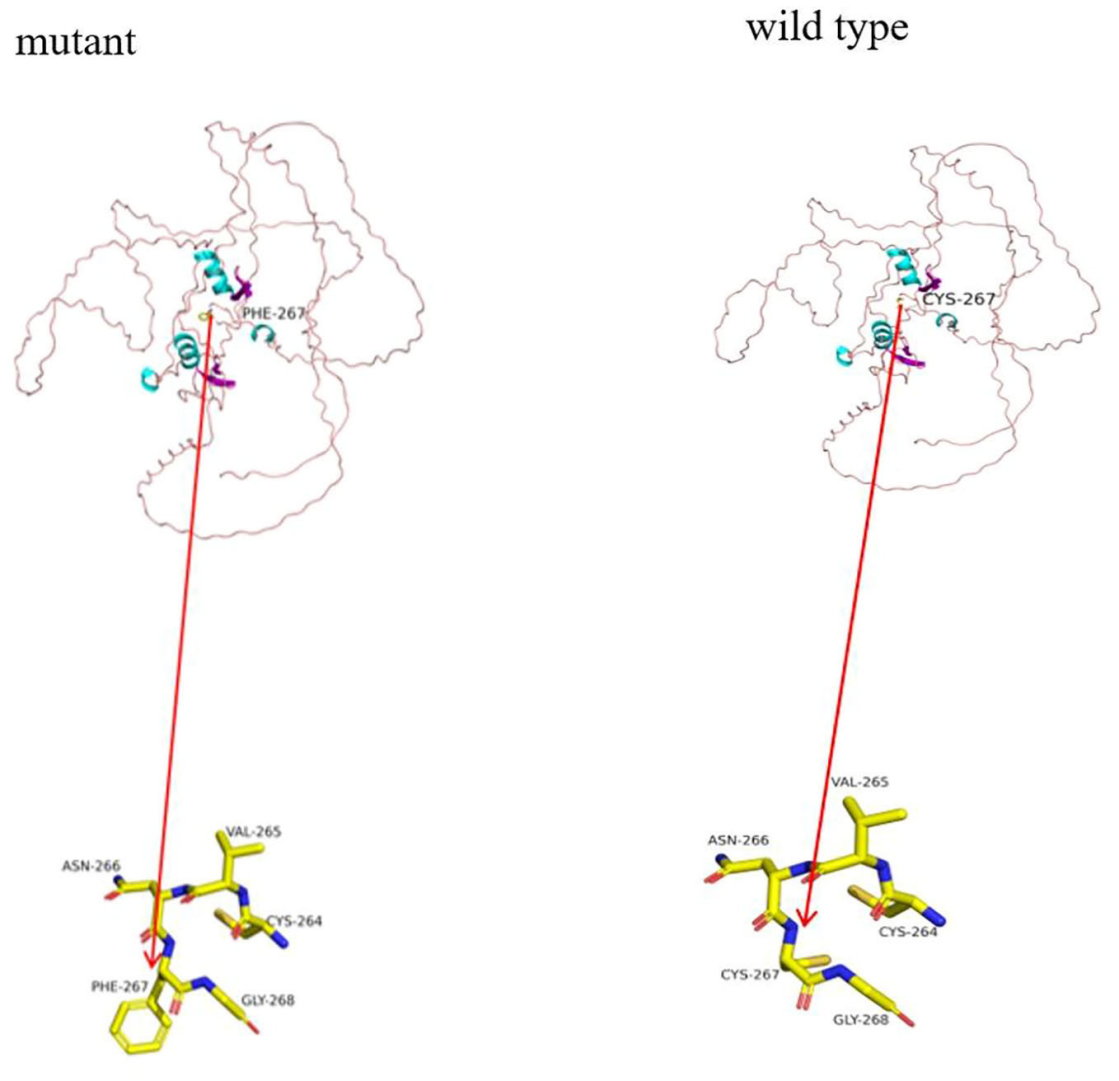
Functional prediction. In the cartoon structure shown in the figure, blue represents α-helices, purple represents β-sheets, and pink loops represent loop structures. The observed hydrogen bonds are depicted as stick structures, with each color representing a different atom: yellow for carbon (C), gray for hydrogen (H), blue for nitrogen (N), red for oxygen (O), and orange for sulfur (S).

Functional prediction (see **Figure 3**): The GATA3 gene’s wild-type three-dimensional model was obtained from the SWISS-MODEL database (https://swissmodel.expasy.org/) and visualized using PyMOL (https://pymol.org/2/). Structural analysis of the Mut-GATA3 protein showed that the p.C267F mutation led to the substitution of the polar neutral amino acid cysteine with the nonpolar hydrophobic amino acid phenylalanine, without affecting hydrogen bonding.

## 3 Discussion

HDR syndrome is a rare autosomal dominant disorder, with GATA3 currently being the only known gene associated with HDR syndrome. Haploinsufficiency of GATA3 is considered a potential cause of HDR syndrome (7). The diagnosis of HDR syndrome is typically based on the identification of clinical symptoms, and the detection of haploinsufficiency or submicroscopic deletions on chromosome 10p14 through DNA analysis can help confirm the diagnosis (8).

The clinical manifestations of patients with HDR syndrome are heterogeneous, even among individuals with the same mutation (9). Studies have shown that only 53.7% of patients exhibit the full HDR triad, while the remaining patients display only two or one of the characteristics (2). Sensorineural hearing loss is often the initial manifestation of HDR syndrome and is typically present at birth. The severity of hearing impairment varies but tends to worsen with age (10). GATA3 plays a crucial role in the development of the cochlear anterior sensory domain and spiral ganglion neurons during embryogenesis, affecting all hair cells and supporting cells in the cochlea. This defect can persist from early infancy to adulthood (11). Despite the fact that this patient passed the hearing screening at birth and showed no abnormal electrophysiological changes in the bilateral ABR test, due to the infant’s young age (9 months), a pure tone audiometry (PTA) test could not be fully conducted. Therefore, continuous follow-up and monitoring are still necessary. Hypoparathyroidism manifests as symptomatic or asymptomatic hypocalcemia, including sensory abnormalities, tetany of hands and feet, hand and foot convulsions, seizures, and other symptoms (12, 13). In this case, the patient’s initial diagnosis of hypoparathyroidism was based on the observation of bilateral thumb adduction and palm rigidity, accompanied by rigidity in the right lower limb. These symptoms were alleviated after calcium supplementation. Additionally, the child’s PTH levels remained persistently below the normal range. Previous studies in heterozygous GATA3 gene knockout mice have shown smaller parathyroid glands. Additionally, low calcium-induced hypocalcemia with low vitamin D intake does not increase parathyroid proliferation or PTH secretion (14). These findings align with the observed hypocalcemia and low PTH levels in our clinical observations.

The severity of renal involvement in HDR syndrome can vary, ranging from no renal abnormalities to various conditions such as renal dysplasia, hypoplasia, cystic kidneys, and bladder-ureter reflux. In some cases, patients may progress to late-stage chronic kidney disease, necessitating renal replacement therapy (15). Studies have shown that the prognosis of HDR syndrome depends on the nature and severity of renal disease, with renal developmental abnormalities typically associated with a poorer prognosis (16).In this case, no renal abnormalities were detected in the patient, potentially due to their young age and the kidneys still being in the developmental process without exhibiting apparent abnormalities. However, it is crucial not to relax the monitoring of these clinical symptoms. Ikeuchi et al. (17) reported a case of an HDR patient who initially presented with primary hypoparathyroidism and right sensorineural hearing loss in the neonatal period, but no renal abnormalities were observed at that time. It was not until the patient reached 11 years old that right renal hypoplasia was discovered, despite the absence of evident clinical symptoms. In addition to the typical triad, previous literature has also reported other symptoms, including congenital heart defects, biliary atresia, pituitary hypofunction, genital anomalies, and diabetes (18–22).Therefore, comprehensive evaluation and regular follow-up are essential for managing patients with HDR syndrome to detect and address any potential complications.

The GATA3 protein comprises two N-terminal transactivation domains (TA1, TA2) and two C-terminal zinc finger domains (ZnF1, ZnF2). ZnF2 specifically recognizes and binds to the N-(A/T)GATA(T/G)-C sequence in DNA. ZnF1 stabilizes the binding of ZnF2 to DNA and interacts with various zinc finger proteins (23, 24). Lemos et al. compiled and analyzed data from 124 reported HDR syndrome families (including 177 patients), summarizing the GATA3 gene mutations found within these families. These mutations included 40% frameshift mutations, 23% missense mutations, 14% nonsense mutations, 6% splice site mutations, 1% in-frame deletions or insertions, 15% whole-gene deletions, and 1% gene duplications. There were no apparent mutation hotspots across the entire GATA3 gene, but missense mutations clustered within or near the ZnF1 or ZnF2 domains of the GATA3 gene (2). In this study, p.C267F is a missense mutation where the polar neutral amino acid cysteine is changed to the non-polar hydrophobic amino acid phenylalanine, located within ZnF1, resulting in a decrease in the affinity of the DNA binding site. This site has not been reported before and has important clinical significance for enriching the mutation spectrum of HDR genes.

Symptomatic management is typically the main approach for treating HDR syndrome. The treatment goal for patients with hypoparathyroidism is to alleviate symptoms and maintain serum calcium levels within the normal low range. However, it is important to note that patients with hypoparathyroidism have reduced renal reabsorption of calcium due to low levels of PTH, which makes them more susceptible to hypercalciuria compared to individuals without the condition. Furthermore, HDR syndrome patients have an inherent risk of kidney disease. Therefore, close monitoring and follow-up are necessary, and measures should be taken to prevent iatrogenic kidney stone formation (25, 26). For hearing impairment, the use of conventional hearing aids or cochlear implants can help restore hearing. Early diagnosis and intervention for hearing impairment are crucial (27). Genetic counseling is particularly important for autosomal dominant genetic disorders like HDR syndrome. It is recommended to seek early evaluation by clinical geneticists. Additionally, attention should be given to newborn hearing screening, prenatal ultrasound examinations, and early identification of hearing or renal defects to provide important opportunities for screening, diagnosis, guidance for reproductive choices, and early intervention.

The analysis of this case’s diagnosis and treatment process offers valuable insights for the clinical management of HDR syndrome. Clinicians should exercise caution when diagnosing HDR syndrome in young patients presenting with hypoparathyroidism, but without evident kidney or hearing symptoms. Early evaluation by clinical geneticists is recommended for autosomal dominant genetic disorders, and emphasis should be placed on newborn hearing screening and continuous monitoring of kidney development. Furthermore, the identification of the novel GATA3 gene variant c.800G>T (p.C267F) in this case contributes to the enrichment of the genetic database.

## Data Availability

The original contributions presented in the study are included in the article/supplementary material. Further inquiries can be directed to the corresponding author.
